# Asprosin inhibits macrophage lipid accumulation and reduces atherosclerotic burden by up-regulating ABCA1 and ABCG1 expression via the p38/Elk-1 pathway

**DOI:** 10.1186/s12967-022-03542-0

**Published:** 2022-07-28

**Authors:** Jin Zou, Can Xu, Zhen-Wang Zhao, Shan-Hui Yin, Gang Wang

**Affiliations:** 1 The First Affiliated Hospital, Department of Cardiology, Hengyang Medical School, University of South China, Hengyang, 421001 Hunan People’s Republic of China; 2 Institute of Cardiovascular Disease, Key Lab for Arteriosclerology of Hunan Province, Hunan International Scientific and Technological Cooperation Base of Arteriosclerotic Disease, Hunan Province Cooperative Innovation Center for Molecular Target New Drug Study, Hengyang Medical School, University of South China, Hengyang, 421001 Hunan People’s Republic of China; 3 The First Affiliated Hospital, Department of Neonatology, Hengyang Medical School, University of South China, Hengyang, 421001 Hunan People’s Republic of China

**Keywords:** Asprosin, p38, Elk-1, ABCA1, ABCG1, Atherosclerosis

## Abstract

**Background:**

Asprosin, a newly discovered adipokine, is a C-terminal cleavage product of profibrillin. Asprosin has been reported to participate in lipid metabolism and cardiovascular disease, but its role in atherogenesis remains elusive.

**Methods:**

Asprosin was overexpressed in THP-1 macrophage-derived foam cells and apoE^−/−^ mice using the lentiviral vector. The expression of relevant molecules was determined by qRT-PCR and/or western blot. The intracellular lipid accumulation was evaluated by high-performance liquid chromatography and Oil red O staining. HE and Oil red O staining was employed to assess plaque burden in vivo. Reverse cholesterol transport (RCT) efficiency was measured using [^3^H]-labeled cholesterol.

**Results:**

Exposure of THP-1 macrophages to oxidized low-density lipoprotein down-regulated asprosin expression. Lentivirus-mediated overexpression of asprosin promoted cholesterol efflux and inhibited lipid accumulation in THP-1 macrophage-derived foam cells. Mechanistic analysis revealed that asprosin overexpression activated p38 and stimulated the phosphorylation of ETS-like transcription factor (Elk-1) at Ser383, leading to Elk-1 nuclear translocation and the transcriptional activation of ATP binding cassette transporters A1 (ABCA1) and ABCG1. Injection of lentiviral vector expressing asprosin diminished atherosclerotic lesion area, increased plaque stability, improved plasma lipid profiles and facilitated RCT in apoE^−/−^ mice. Asprosin overexpression also increased the phosphorylation of p38 and Elk-1 as well as up-regulated the expression of ABCA1 and ABCG1 in the aortas.

**Conclusion:**

Asprosin inhibits lipid accumulation in macrophages and decreases atherosclerotic burden in apoE^−/−^ mice by up-regulating ABCA1 and ABCG1 expression via activation of the p38/Elk-1 signaling pathway.

**Supplementary Information:**

The online version contains supplementary material available at 10.1186/s12967-022-03542-0.

## Introduction

Atherosclerosis is a key etiology of cardiovascular disease, including myocardial infarction, unstable angina and ischemic stroke. Macrophages are the most abundant cell type in atherosclerotic lesions and have an essential role during all stages of this disease, from lesion initiation to ultimately rupture of arterial plaques [1]. The transformation of macrophages into lipid-rich foam cells in the subendothelial space, a hallmark of early stage atherosclerotic lesions, is mainly caused by the imbalance between modified lipoprotein uptake and cholesterol efflux [2]. ATP-binding cassette (ABC) transporters A1 (ABCA1) and ABCG1 belong to integral membrane proteins. The major function of ABCA1 is to promote the transport of intracellular free cholesterol (FC) and phospholipids to extracellular lipid-poor apolipoprotein A-I (apoA-I) for forming nascent high-density lipoprotein (HDL) particles [3]. Subsequently, ABCG1 mediates the export of FC to exogenous HDL for further maturation [4]. ABCA1- and ABCG1-dependent cholesterol efflux is the first and rate-limiting step of reverse cholesterol transport (RCT), a process by which excessive cholesterol in arterial wall cells is delivered by HDL to the liver for further metabolism and excretion [5, 6]. Studies from our group and others have demonstrated that astragalin, C1q tumor necrosis factor-related protein 12, fargesin and biochanin A increase RCT efficiency and protect against atherosclerosis in apolipoprotein E-deficient (apoE^−/−^) mice by up-regulating ABCA1 and ABCG1 expression [7–9]. Thus, searching for the novel mechanisms governing expression regulation of these two transporters is of critical importance to develop future therapeutic intervention in atherosclerotic cardiovascular disease.

Asprosin, a C-terminal cleavage product of profibrillin, contains 140 amino acid residues and is encoded by exons 65 and 66 of the *Fibrillin 1* gene. As a novel glucogenic adipokine, asprosin was first discovered by Romere et al. from neonatal progeroid syndrome patients in 2016 [10]. Although white adipose tissue is regarded as a major source of asprosin, other tissues and cells such as placenta, hepatocytes and cardiomyocytes can also produce this adipokine [11, 12]. Asprosin plays an important role in regulating hepatic glucose release [10], insulin secretion [13], appetite [14], and inflammatory response [15]. Of note, a recent study showed that dilated cardiomyopathy patients with lower plasma asprosin levels have an incremental risk for adverse cardiovascular events during the 5 year follow-up [16]. In patients with coronary artery disease, circulating asprosin levels are directly correlated with total cholesterol (TC) and triglycerides (TG) concentrations [17]. Additionally, injection of asprosin-pretreated mesenchymal stromal cells markedly elevates left ventricular ejection fraction and ameliorates myocardial fibrosis four weeks after myocardial infarction in mice [18]. These findings suggest an association of asprosin with lipid metabolism and cardiovascular disease. However, it is largely unknown whether and how asprosin has an effect on ABCA1 and ABCG1 expression and atherosclerosis.

Mitogen-activated protein kinases (MAPKs) are serine-threonine kinases and comprise three members in mammals: extracellular signal-regulated kinases 1/2 (ERK1/2), c-Jun N-terminal kinase (JNK) and p38. Activation of p38 has been shown to regulate macrophage lipid accumulation and plaque formation [19, 20]. ETS-like transcription factor (Elk-1) belong to the ETS-domain family and can be phosphorylated and activated by MAPKs [21]. We have previously demonstrated that heat shock protein 70 decreases ABCA1 and ABCG1 expression by preventing Elk-1 from binding to their promoters in macrophages [22]. Nevertheless, very little is known about the contribution of the p38/Elk-1 signaling cascade to asprosin-regulated ABCA1 and ABCG1expression.

In this study, we found that asprosin expression was significantly down-regulated in THP-1 macrophages treated with oxidized low-density lipoprotein (ox-LDL). Importantly, lentivirus-mediated overexpression of asprosin activated the p38/Elk-1 pathway to promote ABCA1- and ABCG1-dependent cholesterol efflux in THP-1 macrophage-derived foam cells and decrease atherosclerotic plaque burden in apoE^−/−^ mice fed a Western diet. To our knowledge, this is the first to demonstrate a link of asprosin to macrophage lipid accumulation and atherosclerosis development.

## Materials and methods

### Cells, reagents and antibodies

Human monocyte line THP-1 was purchased from American Type Culture Collection (Rockville, MD, USA). Fetal bovine serum (FBS), phorbol-12-myristate acetate (PMA), apoA-I and SB203580 were provided by Sigma-Aldrich (St. Louis, MO, USA). Ox-LDL, acetylated low-density lipoprotein (ac-LDL) and HDL were obtained from Yiyuan Biotechnology (Guangzhou, China). Lentiviral vectors expressing asprosin (LV-Asprosin) or Elk-1^S383A^ (serine 383 was replaced with alanine) were constructed by Genechem (Shanghai, China). Phosphorylation mutant Elk-1^S383A^ was generated by using a QuikChange II site-directed mutagenesis kit (Stratagene, La Jolla, CA, USA) according to the manufacturer’s instructions. The mutation of Ser383 to alanine was confirmed by DNA sequencing. Rabbit antibodies against liver X receptor α (LXRα), proprotein convertase subtilisin/kexin type 9 (PCSK9), CD36, Elk-1, phospho-Elk-1 (p-Elk-1, Ser383), p-Elk-1 (Ser389), p-Elk-1 (Thr417), p38, phospho-p38 (p-38), JNK, phospho-JNK (p-JNK), ERK1/2, phospho-ERK1/2 (p-ERK1/2), ABCA1, ABCG1, histone H3 and β-actin were supplied by Abcam (Cambridge, UK). Rabbit antibody against asprosin (FineTest, Wuhan, China), mouse antibody against scavenger receptor class A (SR-A; R&D systems, Minneapolis, MN, USA), and horseradish peroxidase (HRP)-conjugated goat anti-rabbit or mouse IgG (H + L) (Beyotime, Shanghai, China) were obtained as indicated.

### Cell culture and treatments

THP-1 cells were cultured in RPMI 1640 medium (Solarbio, Beijing, China) containing 2% penicillin–streptomycin and 10% FBS in a humidified atmosphere of 5% CO_2_ at 37 °C. The differentiation of monocytes to macrophages was induced by the addition 160 nM PMA for 24 h. After 48 h of incubation with 50 µg/mL ox-LDL, macrophages were converted to foam cells. THP-1 macrophage-derived foam cells were seeded in 24-well plates (2 × 10^6^ cells/well) and then transduced with empty vector (LV-Mock; Genechem) or LV-Asprosin at a multiplicity of infection of 100 in the presence of 8 mg/mL polybrene for 24 h. Subsequently, the medium was changed to fresh 10% FBS/RPMI 1640 medium. After 48 h culture, the transfection efficiency was evaluated by western blot analysis of asprosin protein expression. To interfere with the p38/Elk-1 signaling pathway, THP-1 macrophage-derived foam cells were treated with 10 μM SB203580 for 6 h or transfected with Elk-1^S383A^ through lentiviral vector for 72 h. Thereafter, cells were transduced with or without LV-Asprosin for an additional 72 h.

### High-performance liquid chromatography (HPLC)

The intracellular contents of TC, FC and cholesterol ester (CE) were detected using the HPLC analysis as previously described [23]. Briefly, THP-1 macrophage-derived foam cells were washed three times with phosphate-buffered saline (PBS) and then broken by an ultrasonic processor (Scientz, Zhejiang, China) on the ice. Cell lysates were centrifugated at 12,000 g for 5 min to collect protein samples, followed by detection of protein concentration using the BCA Protein Assay Kit (Beyotime). Cholesterol was extracted from protein samples by n-hexane–isopropanol (3:2, V/V) and then dissolved in isopropanol. Subsequently, 0.4 U cholesterol oxidase were added to each sample for measuring the FC content. The TC content was examined by using 0.4 U cholesterol oxidase in combination with 0.4 U cholesterol esterase. CE was calculated by subtracting FC from TC.

### Oil red O staining

After transfection with LV-Mock or LV-Asprosin, THP-1 macrophage-derived foam cells were washed with PBS and then fixed in 4% paraformaldehyde solution for 10 min. Cells were stained with 0.5% Oil Red O solution (Solarbio) in the dark at 37 °C for 5 min, destained with 60% isopropanol for 15 s, incubated with hematoxylin for counterstaining, and then photographed at× 400 magnification.

### Detection of NBD-cholesterol efflux

The NBD-cholesterol efflux assay was performed according to our previous report [24]. In brief, THP-1 macrophage-derived foam cells receiving the indicated treatments were incubated in phenol red-free RPMI 1640 medium supplemented with 5 µmol/L NBD-cholesterol (Invitrogen, Carlsbad, CA, USA) for 4 h at 37 °C. After washing three times with PBS, cells were incubated with 25 μg/mL apoA-I and 50 μg/mL HDL as lipid acceptors for 4 h to examine ABCA1- and ABCG1-dependent cholesterol efflux, respectively. The medium was collected, and cells were harvested and then lysed by 0.1% Triton X-100 (Gibco, Carlsbad, CA, USA). The fluorescence-labeled cholesterol in the medium and cell lysates was visualized by an inverted fluorescence microscope (Olympus IX53, Tokyo, Japan). The Image-Pro Plus software (Diagnostic Instruments, USA) was employed to analyze fluorescence intensity. NBD-cholesterol efflux capacity was calculated as the percentage of fluorescence density in the medium to total fluorescence density (medium + cell lysates).

### Detection of interleukin (IL)-1β and IL-6 secretion

After the indicated treatments, cell culture supernatant was collected to detect the levels of IL-1β and IL-6 by using the commercial enzyme-linked immunosorbent assay (ELISA) kits (R&D systems) according to the manufacturer’s instructions. The absorbance at 450 nm was determined by the iMark™ microplate reader (Bio-Rad, Hercules, CA, USA).

### Measurement of Dil-ox-LDL uptake

The uptake of Dil-ox-LDL by THP-1 macrophage-derived foam cells was performed as previously described [9]. In brief, THP-1 macrophage-derived foam cells were transfected with LV-Mock or LV-Asprosin for 72 h, washed twice with PBS, and then incubated with 10 μg/mL Dil-ox-LDL for 4 h at 37 °C. Cells were washed three times with PBS and then visualized by an inverted fluorescence microscope.

### Animals, diets and treatments

Thirty 8-week-old male apoE^−/−^ mice with a C57BL/6 background were provided by Cavens lab animal (Jiangsu, China) and maintained under a specific-pathogen free condition. These mice were randomly divided into LV-Mock group and LV-Asprosin group, with 15 animals in each group. All mice were fed a Western diet (0.3% cholesterol, 21% fat; Research Diets) for 12 weeks to establish an atherosclerosis model. During this process, LV-Mock or LV-Asprosin (5 × 10^7^ TU/mouse) were injected into the mice via the tail vein once 3 weeks. At the end of the study, the mice were weighed, anesthetized, and then euthanized to collect the blood, aortas and hearts for further analyses. The animal protocols were reviewed and approved by the Animal Care and Use Committee of the First Affiliated Hospital of University of South China.

### Atherosclerotic lesion analyses

The hearts and aortic tissues were immediately isolated from the ascending aorta to the ileal bifurcation and fixed in 4% paraformaldehyde solution after apoE^−/−^ mice were sacrificed. The adventitia was thoroughly cleaned under a dissecting microscope. The aortas were unfolded along the longitudinal axis, stained with 0.5% Oil Red O solution for 30 min and then photographed using a digital camera. The atherosclerotic burden was calculated as the percentage of Oil Red O-positive area to total aorta area. After washing with PBS, the hearts with upper aortic root were embedded in optimal cutting temperature compound (Sakura Finetek, Torrance, CA, USA) and then serially sectioned (8 μm thickness) throughout three aortic valves. Subsequently, the snap-frozen cross-sections were stained with hematoxylin and eosin (HE), Oil Red O and Masson Trichrome for quantification of lesion area, lipid deposition and collagen content using the Image-Pro Plus 6.0 software.

### Detection of plasma lipid and PCSK9 levels

Blood samples were collected from the retro-orbital plexus of apoE^−/−^ mice at the end of experiments and then centrifuged at 800 g for 10 min to isolate the plasma. The levels of TC, low density lipoprotein cholesterol (LDL-C), HDL cholesterol (HDL-C) and TG in plasma were measured by using the commercially available enzymatic kits (Biosino, Beijing, China). Additionally, plasma PCSK9 levels were detected by using ELISA (R&D systems) according to the manufacturer’s protocol.

### RCT assay

The RCT assay was conducted as we had described previously [8]. J774 macrophages were loaded with 5 μCi/mL [^3^H]-cholesterol plus 50 μg/mL ac-LDL for 48 h and then suspended in Dulbecco's Modified Eagle Medium (Gibco). Subsequently, J774 macrophages labeled with [^3^H]-cholesterol (4.5×10^6^ cells/mouse) were intraperitoneally injected into apoE^−/−^ mice (n = 5 in each group). Plasma samples were taken at 6, 24, and 48 h after injection. The feces were continuously collected until 48 h, and hepatic tissue specimens were obtained at 48 h. The radioactivity of [^3^H]-cholesterol in the plasma, liver and feces was analyzed by a liquid scintillation counter. The efficiency of in vivo RCT was calculated as the percentage of radioactivity in the plasma, liver or feces to total radioactivity injected at baseline.

### RNA isolation and quantitative real-time polymerase chain reaction (qRT-PCR)

Total RNA was extracted from cultured cells and mouse aortas using a TRIzol kit (Invitrogen). The obtained RNA was converted to cDNA using a Superscript First-Strand cDNA Synthesis Kit (Invitrogen). Thereafter, qRT-PCR was performed by using SYBR Green Real-Time PCR Master Mix (Promega) on an ABI 7900HT Fast Real-Time PCR System (Applied Biosystems, Foster City, CA, USA). The primers used in this study were designed and synthesized by Sangon Biotech (Shanghai, China). Their sequences are presented in (Additional file 2: Table S1). The relative expression levels of target genes were determined by the 2^−ΔΔ^Ct method. Glyceraldehyde 3-phosphate dehydrogenase (GAPDH) was used as an internal control.

### Protein extraction and western blot

The cultured cells and mouse aortas were lysed by the RIPA buffer containing 0.1 mmol/L phenylmethanesulfonyl fluoride (Beyotime). The cytoplasmic and nuclear proteins were extracted by the corresponding protein extraction kits (Beyotime). The protein concentration was quantified by a BCA Protein Assay Kit. The protein samples were subjected to SDS-PAGE and then transferred to polyvinylidene difluoride membranes (Millipore, Billerica, MA, USA). After blockade in 5% skim milk for 1 h at room temperature, the membranes were incubated with primary antibodies against asprosin (FNab09797, 1:1000), LXRα (ab176323, 1:2000), PCSK9 (ab95478, 1:1000), CD36 (ab133625, 1:500), SR-A (AF1797, 1:1000), Elk-1 (ab11847, 1:500), p-Elk-1 (Ser383; ab34270, 1:300), p-Elk-1 (Ser389; ab28818, 1:300), p-Elk-1 (Thr417; ab28817, 1:300), p38 (ab223619, 1:500), p-p38 (ab32557, 1:1000), JNK (ab112501, 1:500), p-JNK (ab124956, 1:1000), ERK1/2 (ab17942, 1:1000), p-ERK1/2 (ab223500, 1:400), ABCA1 (ab7360, 1:200), ABCG1 (ab52617, 1:1000), histone H3 (ab176842, 1:3000) or β-actin (ab115777, 1:3000) overnight at 4 °C. Subsequently, the membranes were incubated and reacted with HRP-conjugated secondary antibodies (1:3000) for 2 h at room temperature. The immunoreactive bands were visualized using the BeyoECL Plus kit (Beyotime) on Tanon 5500 (Shanghai, China). β-actin was used as an internal control.

### Statistical analysis

All results are presented as the mean ± standard deviation (SD). An unpaired two-tailed Student’s *t*-test was applied to compare the differences between two groups. The differences among ≥ three groups were analyzed using one-way ANOVA followed by Tukey’s multiple comparison test. A value of *P* < 0.05 was thought to be statistically significant. The data were statistically analyzed and graphed using the GraphPad Prism 8.0 software (San Diego, CA, USA).

## Results

### Asprosin inhibits lipid accumulation in THP-1 macrophage-derived foam cells

**Fig. 1:**
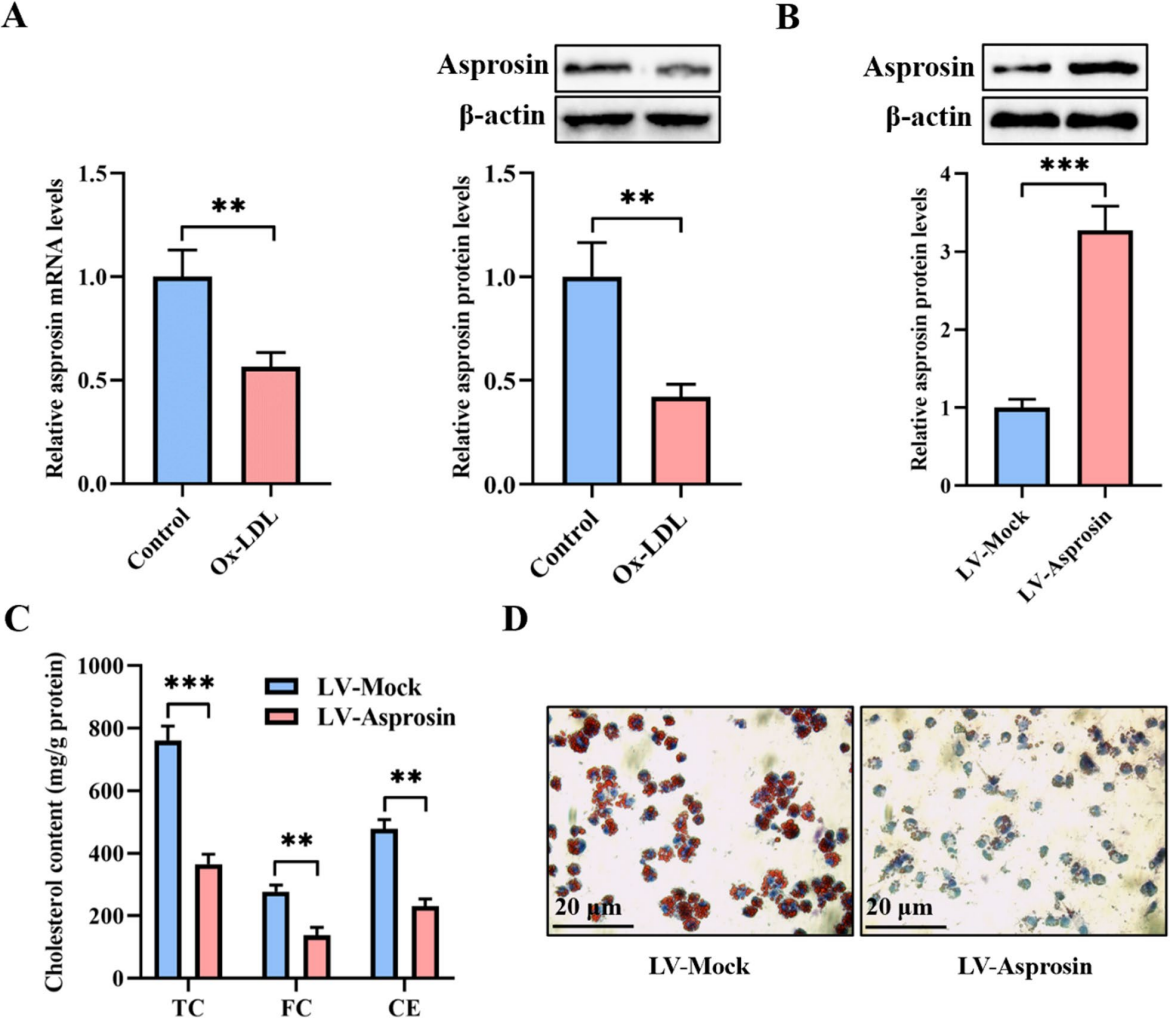
Asprosin prevents macrophages from lipid accumulation. **A** After incubation of THP-1 macrophages with or without 50 µg/mL ox-LDL for 48 h, qRT-PCR and western blot were used to detect asprosin expression (n = 3). **B**–**D** THP-1 macrophage-derived foam cells were transfected with LV-Mock or LV-Asprosin for 72 h (n = 3). **B** Protein samples were immunoblotted with antibodies against asprosin or β-actin. **C** The contents of intracellular TC, FC and CE were measured by HPLC. **D** Representative images of Oil red O staining. Data are expressed as mean ± SD. **P < 0.01, ***P < 0.001

Lipid accumulation in macrophages is regarded as a major driver of atherosclerosis. To clarify whether asprosin plays a role in this process, we first detected its expression using qRT-PCR and western blot in THP-1 macrophages loaded with or without ox-LDL. As shown in Fig. 1A, the mRNA and protein levels of asprosin were significantly decreased in ox-LDL-treated THP-1 macrophages compared with unstimulated cells, indicating a link between asprosin and lipid metabolism. Subsequently, THP-1 macrophage-derived foam cells were transfected with LV-Mock or LV-Asprosin to overexpress asprosin. The transfection efficiency was verified by a marked elevation in asprosin protein levels after LV-Asprosin treatment (Fig. 1B). At the same time, the HPLC analysis showed that asprosin overexpression dramatically reduced the amounts of intracellular TC, FC, and CE (Fig. 1C). Oil Red O has been widely employed to stain neutral lipids, including those present in lipid-rich foam cells in *vitro* and in vivo [25]. The Oil Red O staining results demonstrated that intracellular lipid droplets were significantly decreased in response to LV-asprosin transduction (Fig. 1D). These findings suggest that asprosin plays a protective role in controlling lipid accumulation within THP-1 macrophage-derived foam cells.

### Asprosin increases ABCA1 and ABCG1 expression, promotes cholesterol efflux and inhibits inflammatory response in THP-1 macrophage-derived foam cells

**Fig. 2:**
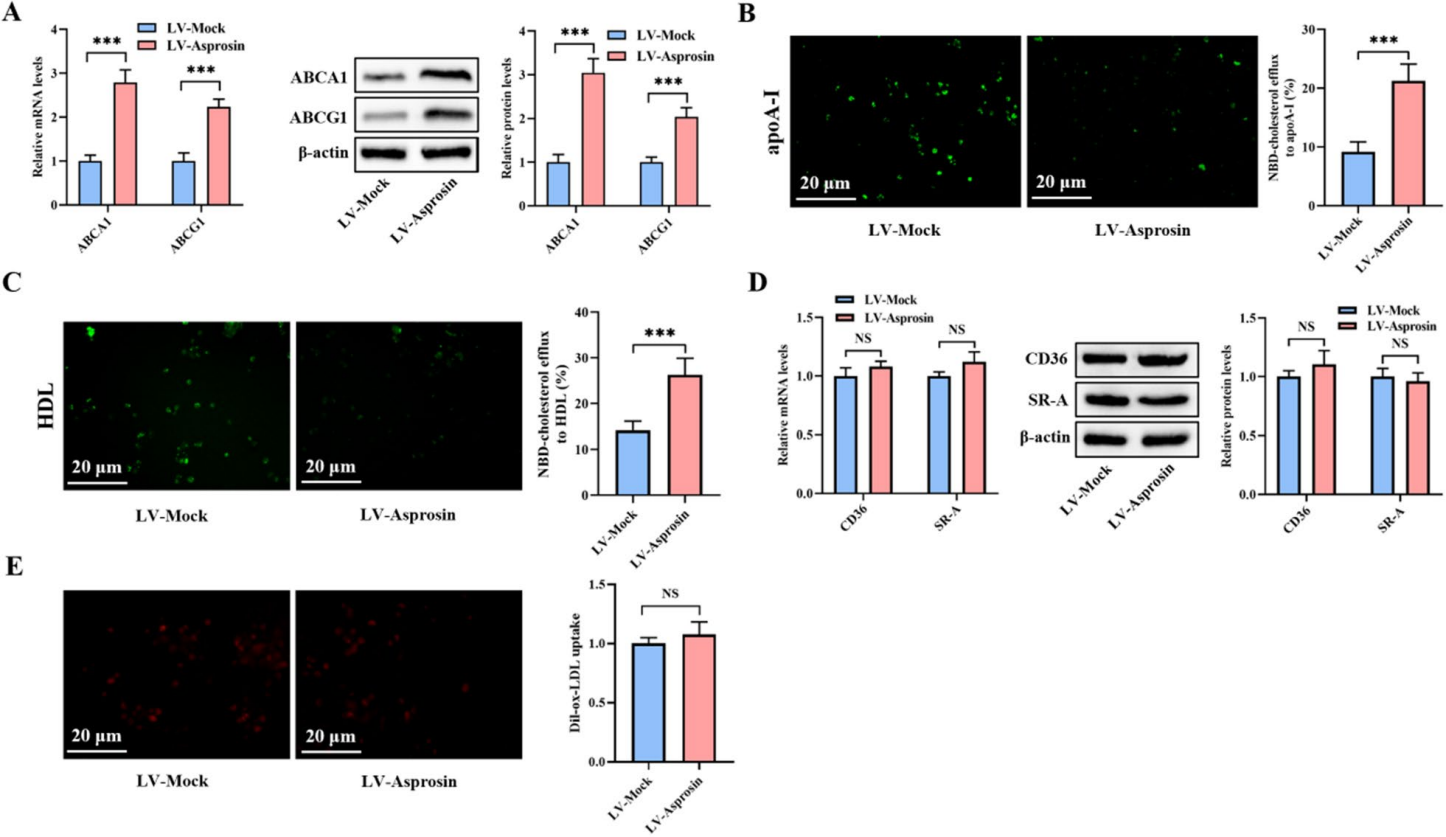
Asprosin promotes ABCA1 and ABCG1 expression and cholesterol efflux from macrophages. **A**–**E** THP-1 macrophage-derived foam cells were transduced with LV-Mock or LV-Asprosin for 72 h (n = 3). **A** The expression of ABCA1 and ABCG1 was determined by qRT-PCR and western blot. **B**, **C** Representative fluorescent images of NBD-cholesterol burden and quantitative analyses of cholesterol efflux to apoA-I and HDL. **D** The qRT-PCR and western blot assays of CD36 and SR-A expression. **E** Representative fluorescent images and quantification of Dil-ox-LDL uptake. Data presented are mean ± SD. ***P < 0.001. NS indicates not significant

It is well established that decreased cholesterol efflux mediated by ABCA1 and ABCG1 is a major cause leading to intracellular lipid accumulation [26]. To reveal the underlying mechanism by which asprosin protects against macrophage lipid accumulation, we examined the effect of asprosin on these two transporter expression and cholesterol efflux capacity in THP-1 macrophage-derived foam cells. As expected, asprosin overexpression augmented the mRNA and protein levels of ABCA1 and ABCG1 (Fig. 2A), with an accompanying increase in NBD-cholesterol efflux to apoA-I (Fig. 2B) and HDL (Fig. 2C). CD36 and SR-A belong to the members of scavenger receptor family and play a central role in mediating the internalization of modified lipoproteins by macrophages [27]. In addition to decreased cholesterol efflux, increased cholesterol influx contributes to intracellular lipid accumulation [28]. However, we found no significant difference in CD36 and SR-A expression in THP-1 macrophage-derived foam cells transfected with LV-Asprosin compared with LV-Mock (Fig. 2D). Consistently, no change was detected in Dil-ox-LDL uptake between LV-Asprosin group and LV-Mock group (Fig. 2E), thereby ruling out the possibility that asprosin-induced prevention of macrophage lipid accumulation is caused by decreased cholesterol uptake. Collectively, these observations suggest that asprosin suppresses lipid deposition in THP-1 macrophage-derived foam cells by stimulating ABCA1- and ABCG1-dependent cholesterol export.

In addition to promoting intracellular cholesterol release, ABCA1 and ABCG1 play an important role in inhibiting inflammatory response [29, 30]. The above studies have demonstrated that asprosin functions as a positive regulator of ABCA1 and ABCG1 expression. We next evaluated the impact of asprosin on pro-inflammatory cytokine production and secretion in THP-1 macrophage-derived foam cells. Transfection with LV-Asprosin dramatically down-regulated the mRNA expression of IL-1β and IL-6 (Additional file 1: Fig. S1A). Asprosin overexpression also diminished the levels of IL-1β and IL-6 in the cell culture supernatant (Additional file 1: Fig. S1B). These data suggest that asprosin contributes to alleviation of macrophage inflammatory response.

### Asprosin up-regulates ABCA1 and ABCG1 expression by phosphorylating Elk-1 at Ser383

**Fig. 3:**
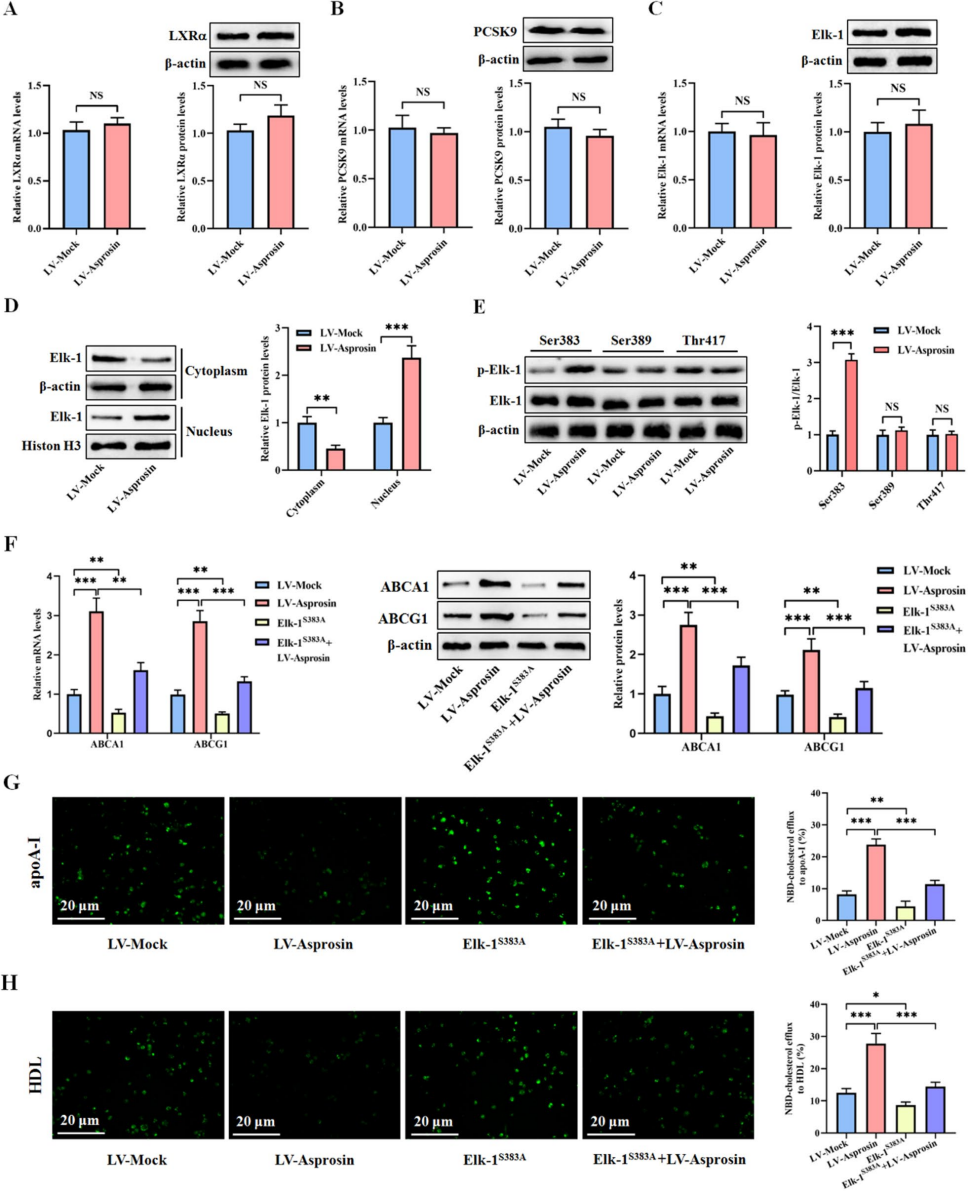
Elk-1 phosphorylation at Ser383 is involved in asprosin-induced up-regulation of ABCA1 and ABCG1 expression. **A–E** THP-1 macrophage-derived foam cells were treated with LV-Mock or LV-Asprosin for 72 h (n = 3). **A** Detection of LXRα expression using qRT-PCR and western blot. **B** The expression of PCSK9 was measured by using qRT-PCR and western blot. **C** The mRNA and protein levels of Elk-1 were determined by qRT-PCR and western blot, respectively. **D** Western blot analysis of Elk-1 protein expression in the cytoplasm and nucleus. **E** Measurement of Elk-1 phosphorylation at Ser383, Ser389 and Thr417 by western blot. **F–H** THP-1 macrophage-derived foam cells were transfected with Elk-1^S383A^ through lentiviral vector for 72 h, followed by treatment with or without LV-Asprosin for another 72 h (n = 3). **F** The qRT-PCR and western blot analyses of ABCA1 and ABCG1 expression. **G**, **H** Representative fluorescent images of NBD-cholesterol burden along with quantitative analyses of cholesterol efflux mediated by apoA-I and HDL. Data shown are mean ± SD. *P < 0.05, **P < 0.01, ***P < 0.001. NS indicates not significant

LXRα, a member of the nuclear receptor superfamily, is thought to be the most important transcription factor to induce ABCA1 and ABCG1 expression [31]. Unexpectedly, we found no difference in LXRα mRNA and protein expression in THP-1 macrophage-derived foam cells treated with LV-Asprosin compared with LV-Mock (Fig. 3A). This finding indicates that LXRα is not implicated in asprosin-enhanced ABCA1 and ABCG1 expression. Interestingly, PCSK9, a subtilisin family-serine protease predominantly expressed in the liver, has been reported to inhibit ABCA1 expression in macrophages [32]. However, asprosin overexpression had no effect on the mRNA and protein levels of PCSK9 in THP-1 macrophage-derived foam cells (Fig. 3B). Thus, like LXRα, PCSK9 is not involved in asprosin-induced up-regulation of ABCA1 and ABCG1 expression. Our previous study has identified Elk-1 as a transcription factor of ABCA1 and ABCG1 [22]. We hypothesized that the stimulatory effect of asprosin on ABCA1 and ABCG1 expression is likely mediated by Elk-1. To test this hypothesis, we measured Elk-1 expression in THP-1 macrophage-derived foam cells and observed similar changes of Elk-1 mRNA and protein levels between LV-Asprosin group and LV-Mock group (Fig. 3C). Given that the transcriptional activity of Elk-1 is dependent on its translocation from the cytoplasm to the nucleus [21], We extracted cytoplasmic and nuclear proteins to determine the distribution of Elk-1 in THP-1 macrophage-derived foam cells. Our results showed that asprosin overexpression increased Elk-1 expression in the nucleus but decreased its expression in the cytoplasm (Fig. 3D), suggesting that asprosin can promote the nuclear translocation of Elk-1. The phosphorylation of Elk-1 at Ser383, Ser389 or Thr417 drives it to enter the nucleus so as to exert a transcriptional activation effect [33–35]. Next, we examined p-Elk-1 levels at these phosphorylated sites. Interestingly, asprosin overexpression elevated p-Elk-1 levels at Ser383 but not Ser389 and Thr417 (Fig. 3E). To determine the role of Ser383 in asprosin-induced enhancement of ABCA1 and ABCG1 expression and cholesterol efflux, we constructed a phosphorylation mutant Elk-1^S383A^, which was transfected into THP-1 macrophage-derived foam cells prior to LV-Asprosin treatment. Mutation to alanine of Ser383 abrogated the effect of LV-Asprosin on ABCA1 and ABCG1 expression (Fig. 3F) as well as NBD-cholesterol efflux to apoA-I (Fig. 3G) and HDL (Fig. 3H). Taken together, these data support the concept that asprosin phosphorylates Elk-1 at Ser383 to induce transcriptional activation of ABCA1 and ABCG1 and promote subsequent cholesterol efflux.

### p38 is required for asprosin-induced increase in Elk-1 phosphorylation and ABCA1 and ABCG1 expression

**Fig. 4:**
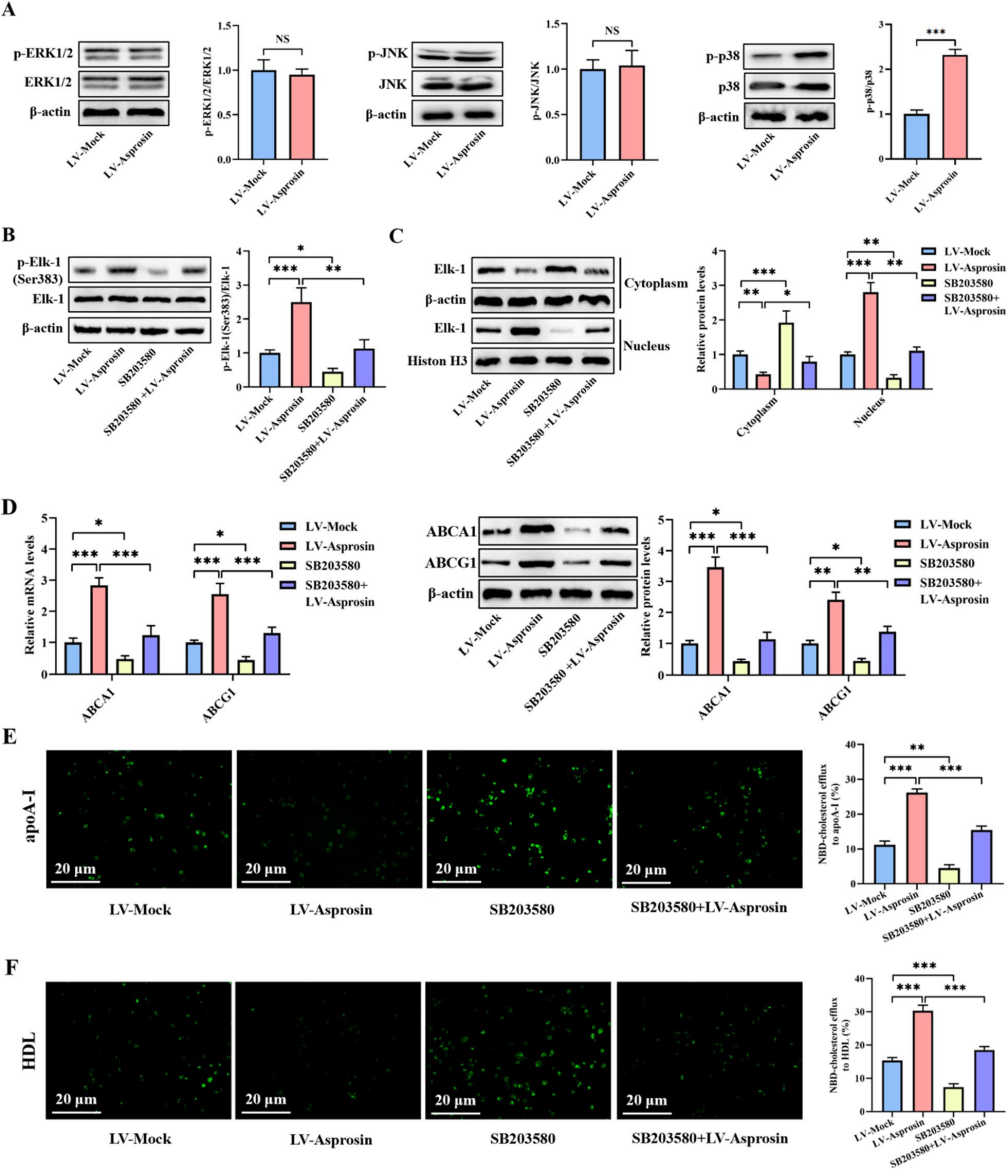
Asprosin stimulates Elk-1 phosphorylation and ABCA1 and ABCG1 expression by activating p38. **A** After 72 h of transfection with LV-Mock or LV-Asprosin, cell lysates were immunoblotted with indicated antibodies (n = 3). **B–F** THP-1 macrophage-derived foam cells were treated with SB203580 for 6 h and then transfected with or without LV-Asprosin for an additional 72 h (n = 3). **B** Detection of Elk-1 phosphorylation at Ser383 using western blot. **C** Western blot analysis of nuclear and cytoplasmic Elk-1 protein expression. **D** Measurement of ABCA1 and ABCG1 expression by qRT-PCR and western blot. **E**, **F** Representative fluorescent images of NBD-cholesterol burden and quantification of cholesterol efflux to apoA-I and HDL. Data represent mean ± SD. *P < 0.05, **P < 0.01, ***P < 0.001. NS indicates not significant

Elk-1 can be activated by three classes of MAPKs: ERK1/2, JNK and p38 [36, 37]. We wondered whether these kinases play a role in the regulation of Elk-1 phosphorylation and ABCA1 and ABCG1 expression by asprosin. To this end, we first treated THP-1 macrophage-derived foam cells with LV-Mock or LV-Asprosin. As illustrated in Fig. 4A, asprosin overexpression markedly promoted the phosphorylation of p38 but not ERK1/2 and JNK, suggesting that asprosin functions an activator of p38. Subsequently, THP-1 macrophage-derived foam cells were incubated with p38 specific inhibitor SB203580, followed by transfection with LV-Asprosin. Pretreatment with SB203580 reduced the effect of LV-asprosin on Elk-1 phosphorylation at Ser383 (Fig. 4B) and nuclear translocation (Fig. 4C). Also, the promotive effect of LV-Asprosin on ABCA1 and ABCG1 expression and NBD-cholesterol efflux was significantly reversed by SB203580 (Fig. 4D-F). These results indicate that asprosin stimulates Elk-1 phosphorylation at Ser383 and promotes ABCA1- and ABCG1-dependent cholesterol release by activating p38 in THP-1 macrophage-derived foam cells.

### Asprosin mitigates atherosclerosis in apoE^−/−^ mice

**Fig. 5:**
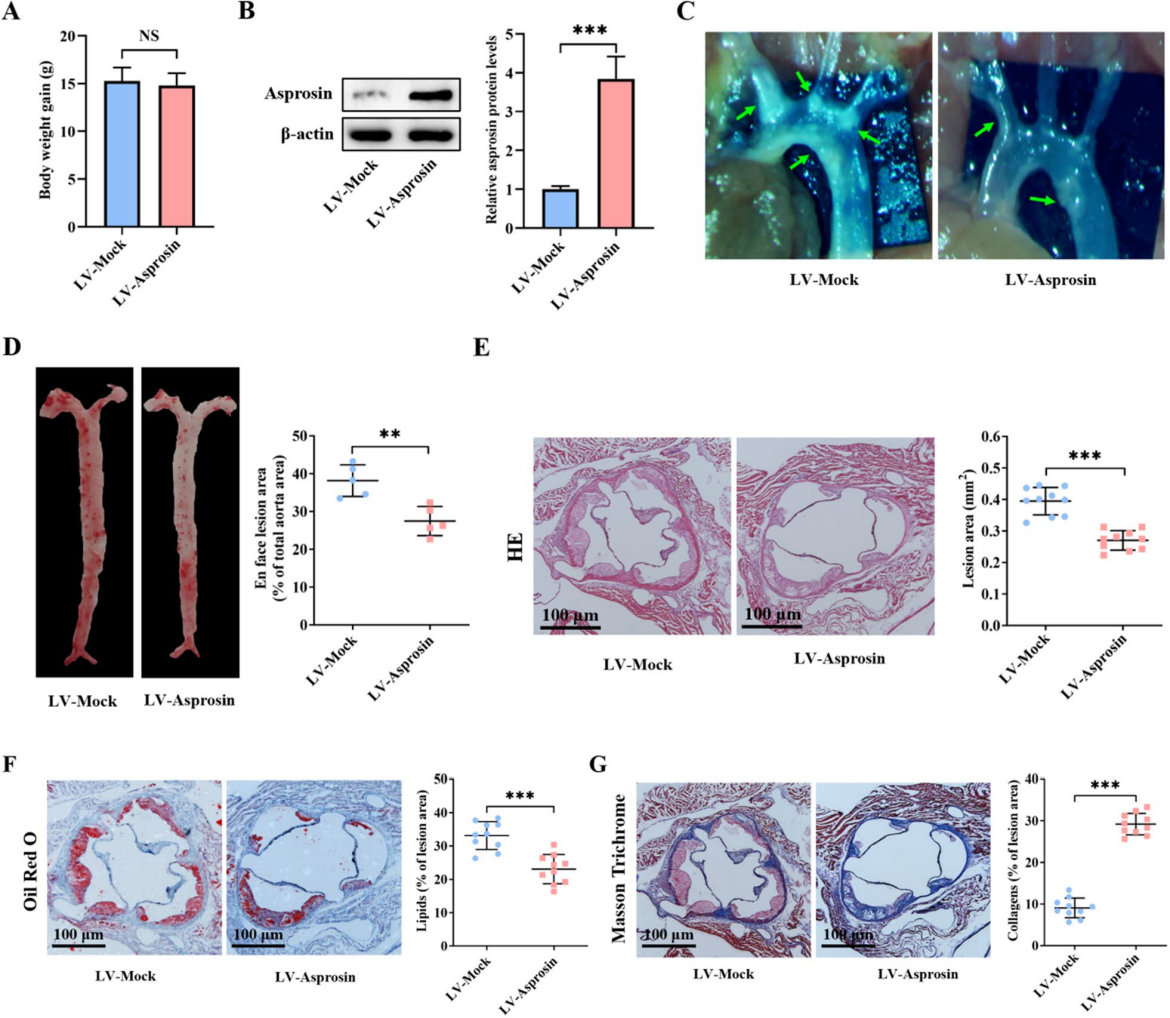
Asprosin inhibits the development of atherosclerosis. **A–G** ApoE^−/−^ mice were fed a Western diet for 12 weeks and simultaneously underwent tail vein injection of LV-Mock or LV-Asprosin once three weeks (n = 15 mice per group). **A** Comparison of body weight gain (n = 15 mice per group). **B** Western blot analysis of asprosin protein expression in the aortas (n = 5 mice per group). **C** Representative images of aortic arch regions with white plaques (green arrows). **D** Enface analysis of atherosclerotic lesion area using Oil Red O staining (n = 5 mice per group). **E–G** The aortic valve cross-sections were stained with HE, Oil Red O and Masson Trichrome to evaluate lesion area, lipid deposition and collagen content, respectively (n = 10 mice per group). The results are shown as the mean ± SD. **P < 0.01, ***P < 0.001. NS indicates not significant

To observe the influence of asprosin on atherosclerotic lesion formation, apoE^−/−^ mice were fed a Western diet for 12 weeks and simultaneously received tail vein injection of LV-Mock or LV-Asprosin. At the end of the study, no significant difference was found in weight gain between LV-Mock and LV-Asprosin mice (Fig. 5A). The western blot results showed that LV-Asprosin mice had a 3.84-fold increase in asprosin protein expression in the aortas as compared to controls (Fig. 5B). This finding suggests a higher transfection efficiency of LV-Asprosin in vivo. Importantly, asprosin overexpression significantly diminished the atherosclerotic plaque burden in the aortic arch regions (Fig. 5C) as well as Oil Red O-positive lesion area in whole enface aortas (Fig. 5D). Consistently, LV-Asprosin mice had a marked reduction in cross-sectional lesion area, as evidenced by HE staining (Fig. 5E). The Oil Red O staining of aortic valve cross-sections demonstrated that atherosclerotic plaques in LV-Asprosin mice had a significant decrease in Oil Red O-positive area compared with LV-Mock mice (Fig. 5F), indicating that asprosin overexpression contributes to alleviation of lesional lipid deposition. The Masson Trichrome staining further showed that collagen content within the plaques was higher in LV-Asprosin mice than LV-Mock mice (Fig. 5G), indicating that asprosin overexpression promotes the shift toward a more stable plaque phenotype. These in vivo data suggest that asprosin exerts an atheroprotective effect.

### Asprosin improves plasma lipid profiles and promotes RCT in apoE^−/−^ mice

**Fig. 6:**
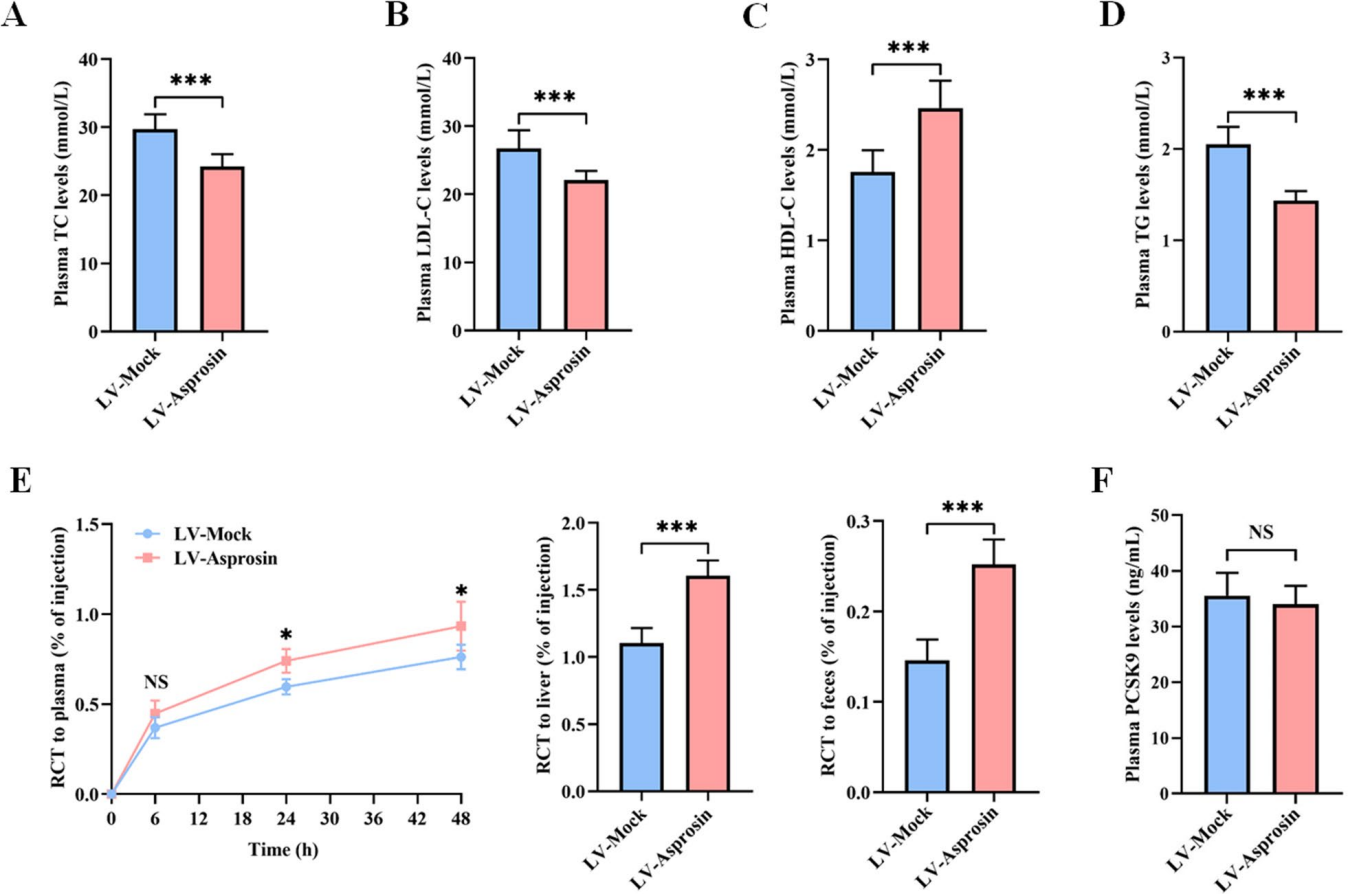
Asprosin ameliorates plasma lipid profile and enhances RCT. **A–D** Circulating levels of TC, LDL-C, HDL-C and TG were measured by the enzymatic methods (n = 10 mice per group). **E** J774 macrophages loaded with ac-LDL and [^3^H]-cholesterol were injected into apo E^−/−^ mice. The radioactivity of [^3^H]-cholesterol in the plasma, liver and feces was determined by a liquid scintillation counter (n = 5 mice per group). **F** ELISA was used to detect plasma PCSK9 levels (n = 10 mice per group). Data are given as the mean ± SD. *P < 0.05, ***P < 0.001. NS indicates not significant

Lipid metabolism disorder is an independent risk factor of atherosclerosis [38]. To gain mechanistical insights into the antiatherogenic action of asprosin, we detected plasma lipid levels and RCT efficiency in apoE^−/−^ mice injected with LV-Mock or LV-Asprosin. Injection of LV-Asprosin significantly increased plasma HDL-C levels but decreased LDL-C, TC and TG levels (Fig. 6A-D). Meanwhile, the radioactivity of [^3^H]-cholesterol in the plasma, liver, and feces was higher in LV-Asprosin group than that in LV-Mock group (Fig. 6E), suggesting that asprosin overexpression contributes to macrophage-to-feces RCT in vivo. In addition, PCSK9 plays an important role in regulating lipid metabolism [39]. Similar to the in vitro data, there was no significant difference in plasma PCSK9 levels between LV-Asprosin group and LV-Mock group (Fig. 6F). These observations reveal that asprosin protects against atherosclerosis by improving plasma lipid profiles and promoting RCT.

### Asprosin stimulates the phosphorylation of p38 and Elk-1 and up-regulates the expression of ABCA1 and ABCG1 in the aortas

**Fig. 7:**
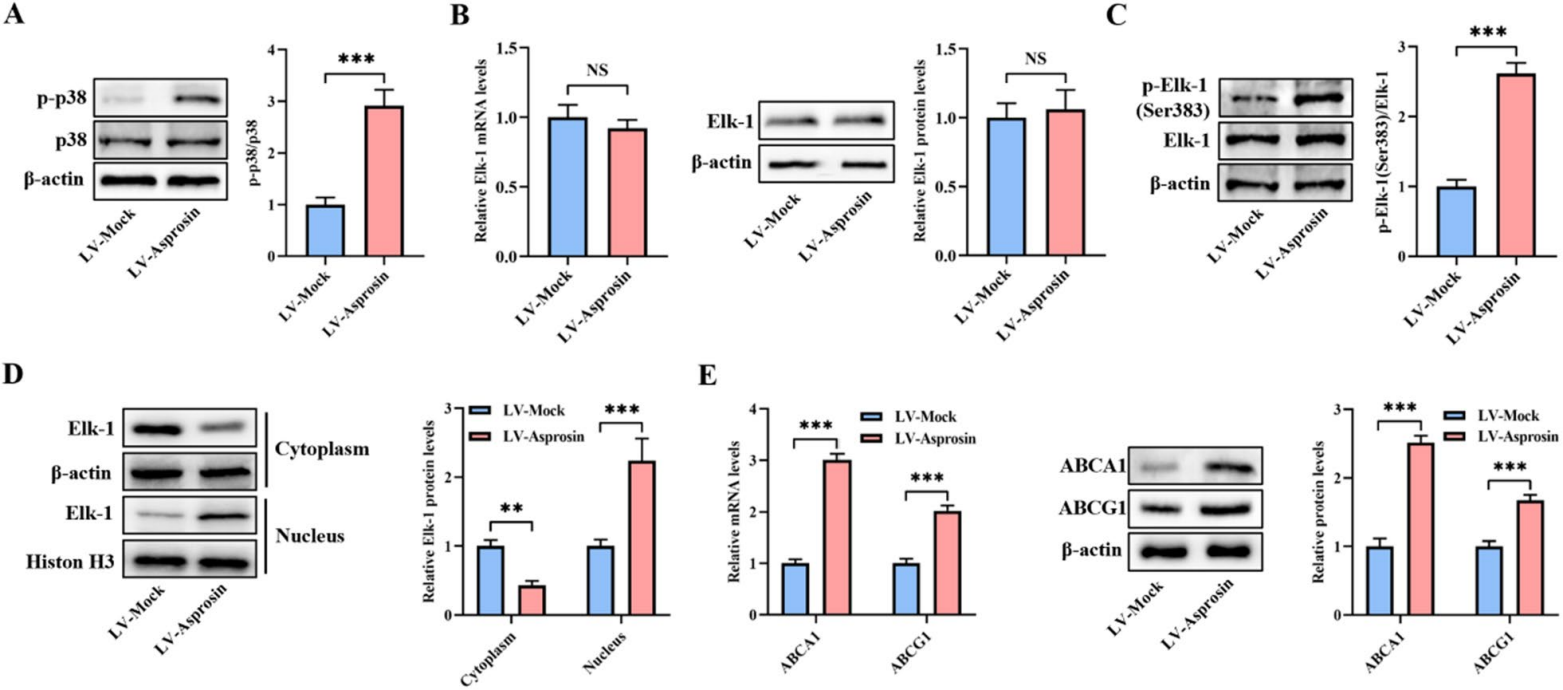
Asprosin promotes p38 and Elk-1 phosphorylation and ABCA1 and ABCG1 expression in the aortas.** A** Protein samples were immunoblotted with antibodies against p-p38, p38 or β-actin in the aortas (n = 5 mice per group). **B** Measurement of aortic Elk-1 expression by qRT-PCR and western blot (n = 5 mice per group). **C** Detection of aortic Elk-1 phosphorylation at Ser383 using western blot (n = 5 mice per group). **D** Western blot analysis of aortic Elk-1 protein levels in the cytoplasm and nucleus (n = 5 mice per group). **E** The qRT-PCR and western blot assays of ABCA1 and ABCG1 expression in the aortas (n = 5 mice per group). The results are shown as the mean ± SD. **P < 0.01, ***P < 0.001. NS indicates not significant

Finally, we isolated the aortas from apoE^−/−^ mice to examine the phosphorylation of p38 and Elk-1 and the expression of Elk-1, ABCA1 and ABCG1. In agreement with the in vitro results, the levels of p-p38 were significantly increased in LV-Asprosin group compared with LV-Mock group (Fig. 7A). Elk-1 mRNA and protein expression in LV-Asprosin mice were not different from LV-Mock mice (Fig. 7B). However, injection of LV-Asprosin augmented p-Elk-1 levels at Ser383 (Fig. 7C) and promoted the translocation of Elk-1 from the cytoplasm to the nucleus (Fig. 7D). Also, asprosin overexpression enhanced the mRNA and protein levels of ABCA1 and ABCG1 (Fig. 7E). These findings suggest that asprosin can activate the p38/Elk-1 signaling pathway, leading to up-regulation of ABCA1 and ABCG1 expression in vivo.

## Discussion

Atherosclerosis constitutes the pathological basis of most cardiovascular disease, which has long been regarded as the major cause of morbidity and mortality in the Western world and is now the first cause of death globally [40]. Adipokines, such as leptin, adiponectin and C1q tumor necrosis factor-related protein 9, have been reported to be involved in the occurrence and development of atherosclerosis [41–43]. As a newly discovered adipokine in 2016, asprosin has multiple effects on central nervous system, peripheral tissues and organs [44]. It is still unclear, however, whether asprosin plays a role in atherogenesis. Here, we observed a significant decrease in asprosin mRNA and protein levels in lipid-laden macrophages. Importantly, we found that apoE^−/−^ mice receiving LV-Asprosin injection developed less lesion area and lipid deposition as well as had increased collagen content. These in vivo data provide strong evidence to support a protective role of asprosin in development of atherosclerosis.

Dyslipidemia, particularly hypercholesterolemia, is thought to be a primary risk factor for atherosclerotic cardiovascular disease. RCT functions as a major approach to eliminate excessive cholesterol from the body and is a critical mechanism for the atheroprotection of HDL [45]. A recent study showed that there is a correlation of serum asprosin levels with TC and TG concentrations in patients with coronary artery disease [17]. Our data demonstrated that overexpression of asprosin in apoE^−/−^ mice promoted macrophage-to-feces RCT together with increased HDL-C levels and decreased LDL-C, TC and TG levels. Thus, improvement of lipid metabolism disorder is a key mechanism by which asprosin protects against atherosclerosis.

Lipid accumulation in macrophages leads to foam cell formation, a hallmark of atherosclerotic lesions throughout all stages of this disease. The formation and retention of lipid-loaded macrophages in the lesion regions exacerbates the disease and accelerates the development of vulnerable plaques [46, 47]. ABCA1 mediates initial transport of FC to apoA-I for nascent HDL biogenesis, while ABCG1 promotes subsequent continued FC efflux to these HDL particles for further maturation [28]. In cholesterol-loaded mouse peritoneal macrophages incubated with diluted human serum, ABCA1 and ABCG1 are responsible for 50% and 20% of cholesterol efflux, respectively [48]. We have previously found that prevention of ABCA1 and ABCG1 expression by angiopoietin-1 or pregnancy-associated plasma protein-A robustly reduces cholesterol export and promotes lipid accumulation in THP-1 macrophage-derived foam cells [49, 50]. Similarly, our results showed that THP-1 macrophage-derived foam cells transfected with LV-Asprosin displayed a significant increase in ABCA1 and ABCG1 expression and subsequent cholesterol efflux, followed up decreased TC, FC, and CE levels. Thus, asprosin suppresses macrophage lipid accumulation and mitigates atherosclerosis by promoting ABCA1- and ABCG1-dependent cholesterol efflux. Atherosclerosis is not only a disorder of lipid metabolism dysregulation, but also a chronic inflammatory disease. Although the major function of ABCA1 and ABCG1 is to promote intracellular cholesterol efflux, there is increasing evidence that these two transporters also exert an anti-inflammatory effect [29, 30]. We found that asprosin overexpression decreased the production and secretion of IL-1β and IL-6 in THP-1 macrophage-derived foam cells. This suggests that inhibition of inflammatory response may be another important mechanism underlying the atheroprotective action of asprosin.

As an ETS transcription factor, Elk-1 can modulate the expression of various genes, which are involved in the regulation of multiple biological functions, including angiogenesis, cell survival and cell differentiation [36, 51, 52]. Overexpression of miR-150 was shown to enhance apoptosis via silencing of Elk-1 in human umbilical vein endothelial cells [53]. We previously reported that Elk-1 can directly bind to the promoters of ABCA1 and ABCG1 for transcriptional activation [22]. Elk-1 functions as a downstream effector of p38. Several lines of evidence have demonstrated that activation of p38 is able to up-regulate the expression of ABCA1 and ABCG1 in macrophages and prostate cancer epithelial cells [54–56]. The transcriptional activity of Elk-1 is dependent on its phosphorylation and subsequent nuclear translocation, which is regulated by the MAPKs [21]. Moreover, the phosphorylation of Ser383 is particularly important for the transactivating effect of Elk-1, because a single mutation of this residue causes a total loss of this effect [57]. In this study, we demonstrated that asprosin overexpression in THP-1 macrophage-derived foam cells activated p38, leading to the increased phosphorylation of Elk-1 at Ser383. Phosphorylated Elk-1 translocated into the nucleus to induce ABCA1 and ABCG1 expression. We also found that blockade of the p38/Elk-1 pathway by SB203580 and mutant Elk-1^S383A^ significantly reversed asprosin-induced enhancement of ABCA1 and ABCG1 expression as well as cholesterol efflux capacity in THP-1 macrophage-derived foam cells. Collectively, these results support the critical role of the p38/Elk-1 signaling in the induction of ABCA1 and ABCG1 expression by asprosin and the subsequent change in cholesterol efflux. It is worth noting that asprosin has been reported to phosphorylate ERK1/2 in mouse mesenchymal stromal cells [18], and JNK in mouse insulinoma MIN6 cells [13]. However, such effects were not present in THP-1 macrophage-derived foam cells transfected with LV-Asprosin. It is thus possible that activation of MAPKs by asprosin may be cell-specific. Future research will be required to test this possibility.

**Fig. 8:**
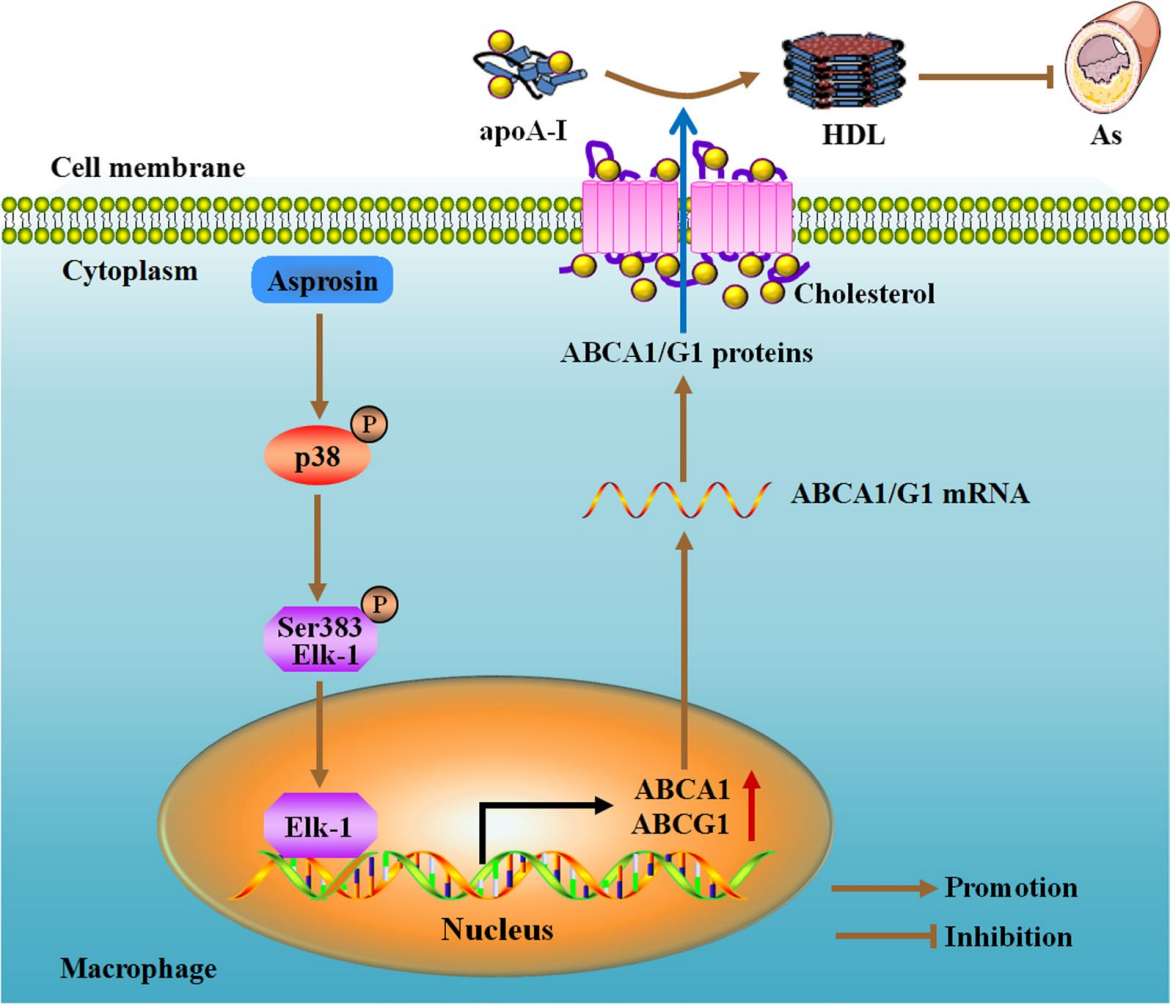
**A** model describing the mechanism for asprosin-induced atheroprotection. Asprosin activates p38 and then phosphorylates Elk-1 at Ser383. After phosphorylation, Elk-1 enters the nucleus to stimulate ABCA1 and ABCG1 transcription. Increased ABCA1 and ABCG1 expression promotes cholesterol efflux from macrophages, leading to acceleration of RCT and alleviation of atherosclerotic plaque burden

In summary, the present study has demonstrated a protective role for asprosin in macrophage lipid accumulation and atherosclerosis development and revealed a novel mechanism underlying the modulation of ABCA1 and ABCG1 expression. Asprosin activates p38 and then phosphorylates Elk-1 at Ser383, thereby promoting Elk-1-dependent ABCA1 and ABCG1 transactivation for enhanced cholesterol efflux from macrophages (Fig. 8). These findings identify the regulation of macrophage asprosin as a novel approach to prevent and treat atherosclerotic cardiovascular disease.

## Supplementary Information

**Additional File 1: Figure S1**. Asprosin suppresses the production and secretion of IL-1β and IL-6. A, B THP-1 macrophage-derived foam cells were transfected with LV-Mock or LV-Asprosin for 72 h (n=3). A Measurement of IL-1β and IL-6 mRNA expression by qRT-PCR. B The cell culture supernatant was collected and then subjected to ELISA for detecting the levels of IL-1β and IL-6. Data are the mean ± SD. **P < 0.01.

**Additional File 2: Table S1**. The primer sequences used in qRT-PCR.

## Data Availability

The data that support the findings of this study are available from the corresponding author upon reasonable request.

## Abbreviations

ABCA1: ATP-binding cassette transporter A1
ABCG1: ATP-binding cassette transporter G1
FC: Free cholesterol
apoA-I: Apolipoprotein A-I
HDL: High-density lipoprotein
RCT: Reverse cholesterol transport
TC: Total cholesterol; TG: triglycerides
MAPKs: Mitogen-activated protein kinases
ERK1/2: Extracellular signal-regulated kinases 1/2
JNK: C-Jun N-terminal kinase
Elk-1: ETS-like transcription factor; ox-LDL: oxidized low-density lipoprotein
FBS: Fetal bovine serum
PMA: Phorbol-12-myristate acetate
ac-LDL: Acetylated low-density lipoprotein
LXRα: Liver X receptor α
PCSK9: Proprotein convertase subtilisin/kexin type 9
HPLC: High-performance liquid chromatography
CE: Cholesterol ester; IL-1β: interleukin-1β
IL-6: Interleukin-6
ELISA: Enzyme-linked immunosorbent assay
LDL-C: Low density lipoprotein cholesterol
GAPDH: Glyceraldehyde 3-phosphate dehydrogenase

## References

[1] Farahi L, Sinha SK, Lusis AJ (2021). Roles of macrophages in atherogenesis. Front Pharmacol.

[2] Li J, Meng Q, Fu Y, Yu X, Ji T, Chao Y, Chen Q, Li Y, Bian H (2021). Novel insights: dynamic foam cells derived from the macrophage in atherosclerosis. J Cell Physiol.

[3] Wang N, Silver DL, Thiele C, Tall AR (2001). ATP-binding cassette transporter A1 (ABCA1) functions as a cholesterol efflux regulatory protein. J Biol Chem.

[4] Kennedy MA, Barrera GC, Nakamura K, Baldan A, Tarr P, Fishbein MC, Frank J, Francone OL, Edwards PA (2005). ABCG1 has a critical role in mediating cholesterol efflux to HDL and preventing cellular lipid accumulation. Cell Metab.

[5] Yu XH, Zhang DW, Zheng XL, Tang CK (2019). Cholesterol transport system: an integrated cholesterol transport model involved in atherosclerosis. Prog Lipid Res.

[6] Chistiakov DA, Melnichenko AA, Myasoedova VA, Grechko AV, Orekhov AN (2017). Mechanisms of foam cell formation in atherosclerosis. J Mol Med (Berl).

[7] Zhao ZW, Zhang M, Wang G, Zou J, Gao JH, Zhou L, Wan XJ, Zhang DW, Yu XH, Tang CK (2021). Astragalin retards atherosclerosis by promoting cholesterol efflux and inhibiting the inflammatory response via upregulating ABCA1 and ABCG1 expression in macrophages. J Cardiovasc Pharmacol.

[8] Wang G, Gao JH, He LH, Yu XH, Zhao ZW, Zou J, Wen FJ, Zhou L, Wan XJ, Tang CK (2020). Fargesin alleviates atherosclerosis by promoting reverse cholesterol transport and reducing inflammatory response. Biochim Biophys Acta Mol Cell Biol Lipids.

[9] Yu XH, Chen JJ, Deng WY, Xu XD, Liu QX, Shi MW, Ren K (2020). Biochanin a mitigates atherosclerosis by inhibiting lipid accumulation and inflammatory response. Oxid Med Cell Longev.

[10] Romere C, Duerrschmid C, Bournat J, Constable P, Jain M, Xia F, Saha PK, Del Solar M, Zhu B, York B (2016). Asprosin, a fasting-induced glucogenic protein hormone. Cell.

[11] Yu Y, He JH, Hu LL, Jiang LL, Fang L, Yao GD, Wang SJ, Yang Q, Guo Y, Liu L (2020). Placensin is a glucogenic hormone secreted by human placenta. EMBO Rep.

[12] Kocaman N, Kuloglu T (2020). Expression of asprosin in rat hepatic, renal, heart, gastric, testicular and brain tissues and its changes in a streptozotocin-induced diabetes mellitus model. Tissue Cell.

[13] Lee T, Yun S, Jeong JH, Jung TW (2019). Asprosin impairs insulin secretion in response to glucose and viability through TLR4/JNK-mediated inflammation. Mol Cell Endocrinol.

[14] Duerrschmid C, He Y, Wang C, Li C, Bournat JC, Romere C, Saha PK, Lee ME, Phillips KJ, Jain M (2017). Asprosin is a centrally acting orexigenic hormone. Nat Med.

[15] Jung TW, Kim HC, Kim HU, Park T, Park J, Kim U, Kim MK, Jeong JH (2019). Asprosin attenuates insulin signaling pathway through PKCdelta-activated ER stress and inflammation in skeletal muscle. J Cell Physiol.

[16] Wen MS, Wang CY, Yeh JK, Chen CC, Tsai ML, Ho MY, Hung KC, Hsieh IC (2020). The role of Asprosin in patients with dilated cardiomyopathy. BMC Cardiovasc Disord.

[17] Moradi N, Fouani FZ, Vatannejad A, Bakhti Arani A, Shahrzad S, Fadaei R (2021). Serum levels of Asprosin in patients diagnosed with coronary artery disease (CAD): a case-control study. Lipids Health Dis.

[18] Zhang Z, Tan Y, Zhu L, Zhang B, Feng P, Gao E, Xu C, Wang X, Yi W, Sun Y (2019). Asprosin improves the survival of mesenchymal stromal cells in myocardial infarction by inhibiting apoptosis via the activated ERK1/2-SOD2 pathway. Life Sci.

[19] Nyandwi JB, Ko YS, Jin H, Yun SP, Park SW, Kim HJ (2021). Rosmarinic acid increases macrophage cholesterol efflux through regulation of ABCA1 and ABCG1 in different mechanisms. Int J Mol Sci.

[20] Park JG, Yoo JY, Jeong SJ, Choi JH, Lee MR, Lee MN, Hwa Lee J, Kim HC, Jo H, Yu DY (2011). Peroxiredoxin 2 deficiency exacerbates atherosclerosis in apolipoprotein E-deficient mice. Circ Res.

[21] Besnard A, Galan-Rodriguez B, Vanhoutte P, Caboche J (2011). Elk-1 a transcription factor with multiple facets in the brain. Front Neurosci.

[22] Zhao ZW, Zhang M, Chen LY, Gong D, Xia XD, Yu XH, Wang SQ, Ou X, Dai XY, Zheng XL (2018). Heat shock protein 70 accelerates atherosclerosis by downregulating the expression of ABCA1 and ABCG1 through the JNK/Elk-1 pathway. Biochim Biophys Acta Mol Cell Biol Lipids.

[23] Yu XH, Deng WY, Chen JJ, Xu XD, Liu XX, Chen L, Shi MW, Liu QX, Tao M, Ren K (2020). LncRNA kcnq1ot1 promotes lipid accumulation and accelerates atherosclerosis via functioning as a ceRNA through the miR-452-3p/HDAC3/ABCA1 axis. Cell Death Dis.

[24] Wang G, Chen JJ, Deng WY, Ren K, Yin SH, Yu XH (2021). CTRP12 ameliorates atherosclerosis by promoting cholesterol efflux and inhibiting inflammatory response via the miR-155-5p/LXRalpha pathway. Cell Death Dis.

[25] Mehlem A, Hagberg CE, Muhl L, Eriksson U, Falkevall A (2013). Imaging of neutral lipids by oil red O for analyzing the metabolic status in health and disease. Nat Protoc.

[26] Chistiakov DA, Bobryshev YV, Orekhov AN (2016). Macrophage-mediated cholesterol handling in atherosclerosis. J Cell Mol Med.

[27] Kunjathoor VV, Febbraio M, Podrez EA, Moore KJ, Andersson L, Koehn S, Rhee JS, Silverstein R, Hoff HF, Freeman MW (2002). Scavenger receptors class A-I/II and CD36 are the principal receptors responsible for the uptake of modified low density lipoprotein leading to lipid loading in macrophages. J Biol Chem.

[28] Yu XH, Fu YC, Zhang DW, Yin K, Tang CK (2013). Foam cells in atherosclerosis. Clin Chim Acta.

[29] Yin K, Liao DF, Tang CK (2010). ATP-binding membrane cassette transporter A1 (ABCA1): a possible link between inflammation and reverse cholesterol transport. Mol Med.

[30] Wojcik AJ, Skaflen MD, Srinivasan S, Hedrick CC (2008). A critical role for ABCG1 in macrophage inflammation and lung homeostasis. J Immunol.

[31] Rigamonti E, Helin L, Lestavel S, Mutka AL, Lepore M, Fontaine C, Bouhlel MA, Bultel S, Fruchart JC, Ikonen E (2005). Liver X receptor activation controls intracellular cholesterol trafficking and esterification in human macrophages. Circ Res.

[32] Adorni MP, Cipollari E, Favari E, Zanotti I, Zimetti F, Corsini A, Ricci C, Bernini F, Ferri N (2017). Inhibitory effect of PCSK9 on Abca1 protein expression and cholesterol efflux in macrophages. Atherosclerosis.

[33] Oh YT, Liu X, Yue P, Kang S, Chen J, Taunton J, Khuri FR, Sun SY (2010). ERK/ribosomal S6 kinase (RSK) signaling positively regulates death receptor 5 expression through co-activation of CHOP and Elk1. J Biol Chem.

[34] Cruzalegui FH, Cano E, Treisman R (1999). ERK activation induces phosphorylation of Elk-1 at multiple S/T-P motifs to high stoichiometry. Oncogene.

[35] Marais R, Wynne J, Treisman R (1993). The SRF accessory protein Elk-1 contains a growth factor-regulated transcriptional activation domain. Cell.

[36] Xu Z, Zhu C, Chen C, Zong Y, Feng H, Liu D, Feng W, Zhao J, Lu A (2018). CCL19 suppresses angiogenesis through promoting miR-206 and inhibiting Met/ERK/Elk-1/HIF-1alpha/VEGF-A pathway in colorectal cancer. Cell Death Dis.

[37] Kim CG, Choi BH, Son SW, Yi SJ, Shin SY, Lee YH (2007). Tamoxifen-induced activation of p21Waf1/Cip1 gene transcription is mediated by early growth response-1 protein through the JNK and p38 MAP kinase/Elk-1 cascades in MDA-MB-361 breast carcinoma cells. Cell Signal.

[38] Lu H, Daugherty A (2015). Atherosclerosis. Arterioscler Thromb Vasc Biol.

[39] Schulz R, Schluter KD (2017). PCSK9 targets important for lipid metabolism. Clin Res Cardiol Suppl.

[40] Roth GA, Huffman MD, Moran AE, Feigin V, Mensah GA, Naghavi M, Murray CJ (2015). Global and regional patterns in cardiovascular mortality from 1990 to 2013. Circulation.

[41] Huang C, Zhang P, Li T, Li J, Liu T, Zuo A, Chen J, Guo Y (2019). Overexpression of CTRP9 attenuates the development of atherosclerosis in apolipoprotein E-deficient mice. Mol Cell Biochem.

[42] Wang Y, Wang X, Guo Y, Bian Y, Bai R, Liang B, Xiao C (2017). Effect of adiponectin on macrophage reverse cholesterol transport in adiponectin-/- mice and its mechanism. Exp Ther Med.

[43] Hoffmann A, Ebert T, Kloting N, Dokas J, Jeromin F, Jessnitzer B, Burkhardt R, Fasshauer M, Kralisch S (2016). Leptin dose-dependently decreases atherosclerosis by attenuation of hypercholesterolemia and induction of adiponectin. Biochim Biophys Acta.

[44] Yuan M, Li W, Zhu Y, Yu B, Wu J (2020). Asprosin: a novel player in metabolic diseases. Front Endocrinol.

[45] Rader DJ, Alexander ET, Weibel GL, Billheimer J, Rothblat GH (2009). The role of reverse cholesterol transport in animals and humans and relationship to atherosclerosis. J Lipid Res.

[46] Back M, Hansson GK (2015). Anti-inflammatory therapies for atherosclerosis. Nat Rev Cardiol.

[47] Libby P, Ridker PM, Maseri A (2002). Inflammation and atherosclerosis. Circulation.

[48] Adorni MP, Zimetti F, Billheimer JT, Wang N, Rader DJ, Phillips MC, Rothblat GH (2007). The roles of different pathways in the release of cholesterol from macrophages. J Lipid Res.

[49] Ou X, Gao JH, He LH, Yu XH, Wang G, Zou J, Zhao ZW, Zhang DW, Zhou ZJ, Tang CK (2020). Angiopoietin-1 aggravates atherosclerosis by inhibiting cholesterol efflux and promoting inflammatory response. Biochim Biophys Acta Mol Cell Biol Lipids.

[50] Tang SL, Zhao ZW, Liu SM, Wang G, Yu XH, Zou J, Wang SQ, Dai XY, Fu MG, Zheng XL (2019). Pregnancy-associated plasma protein-a accelerates atherosclerosis by regulating reverse cholesterol transport and inflammation. Circ J.

[51] Booy EP, Henson ES, Gibson SB (2011). Epidermal growth factor regulates Mcl-1 expression through the MAPK-Elk-1 signalling pathway contributing to cell survival in breast cancer. Oncogene.

[52] Yoshida T, Gan Q, Owens GK (2008). Kruppel-like factor 4, Elk-1, and histone deacetylases cooperatively suppress smooth muscle cell differentiation markers in response to oxidized phospholipids. Am J Physiol Cell Physiol.

[53] Qin B, Shu Y, Xiao L, Lu T, Lin Y, Yang H, Lu Z (2017). MicroRNA-150 targets ELK1 and modulates the apoptosis induced by ox-LDL in endothelial cells. Mol Cell Biochem.

[54] Suzuki K, Kawakami Y, Yamauchi K (2017). Impact of TLR 2, TLR 4-activation on the expression of ABCA1 and ABCG1 in raw cells. Ann Clin Lab Sci.

[55] Chang YC, Lee TS, Chiang AN (2012). Quercetin enhances ABCA1 expression and cholesterol efflux through a p38-dependent pathway in macrophages. J Lipid Res.

[56] Trasino SE, Kim YS, Wang TT (2009). Ligand, receptor, and cell type-dependent regulation of ABCA1 and ABCG1 mRNA in prostate cancer epithelial cells. Mol Cancer Ther.

[57] Gille H, Strahl T, Shaw PE (1995). Activation of ternary complex factor Elk-1 by stress-activated protein kinases. Curr Biol.

