# Supplementary figures and images for: Asprosin inhibits macrophage lipid accumulation and reduces atherosclerotic burden by up-regulating ABCA1 and ABCG1 expression via the p38/Elk-1 pathway
Source: J Transl Med. 2022 Jul 28;20:337. doi: 10.1186/s12967-022-03542-0 (PMC9331044; doi:10.1186/s12967-022-03542-0)

**Additional file 1: Fig.S1**


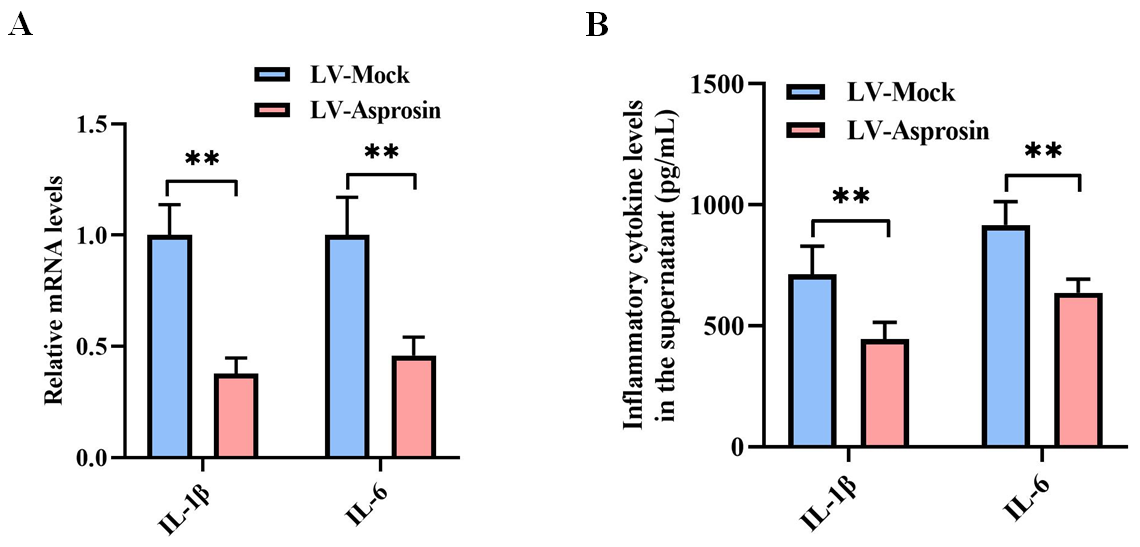

Supplement: Supplementary file 1 — Additional File 1: Figure S1. Asprosin suppresses the production and secretion of IL-1β and IL-6. A, B THP-1 macrophage-derived foam cells were transfected with LV-Mock or LV-Asprosin for 72 h (n=3). A Measurement of IL-1β and IL-6 mRNA expression by qRT-PCR. B The cell culture supernatant was collected and then subjected to ELISA for detecting the levels of IL-1β and IL-6. Data are the mean ± SD. **P < 0.01. [file 12967_2022_3542_MOESM1_ESM.docx]
