# Supplementary material for: Asprosin inhibits macrophage lipid accumulation and reduces atherosclerotic burden by up-regulating ABCA1 and ABCG1 expression via the p38/Elk-1 pathway
Source: J Transl Med. 2022 Jul 28;20:337. doi: 10.1186/s12967-022-03542-0 (PMC9331044; doi:10.1186/s12967-022-03542-0)
Supplement: Supplementary file 2 — Additional File 2: Table S1. The primer sequences used in qRT-PCR. [file 12967_2022_3542_MOESM2_ESM.docx]

**Additional file 1: Table S1**. The primer sequences used in qRT-PCR

| Genes | Sequences |
| --- | --- |
| Human asprosin | 5′-ATGGCTCGTGTAGGACGCTA-3′ (forward) |
|  | 5′-GCCTCGGGGATTTGTCTCTG-3′ (reverse) |
| Human IL-1β | 5′-CCACAGACCTTCCAGGAGAATG-3′ (forward) |
|  | 5′-GTGCAGTTCAGTGATCGTACAGG-3′ (reverse) |
| Human IL-6 | 5′-AGACAGCCACTCACCTCTTCAG-3′ (forward) |
|  | 5′-TTCTGCCAGTGCCTCTTTGCTG-3′ (reverse) |
| Human LXRα | 5′-CCTTCAG AACCCACAGAGATCC-3′ (forward) |
|  | 5′-ACGCTGCATAGCTCGTTCC-3′ (reverse) |
| Human PCSK9 | 5′-TCCACGCTTCCTGCTGCCAT-3′ (forward) |
|  | 5′-CAGGCAGTCAGGGTCCAGCC-3′ (reverse) |
| Human Elk-1 | 5′-TCCCTGCTTCCTACGCATACA-3′ (forward) |
|  | 5′-GCTGCCACTGGATGGAAACT-3′ (reverse) |
| Human ABCA1 | 5′-ACCCACCCTAT GAACAACATGA-3′ (forward) |
|  | 5′-GAGTCGGGTAACGGAAACAGG-3′ (reverse) |
| Mouse ABCA1 | 5′-GGGTGGTGTTCTTCCTCATTAC-3′ (forward) |
|  | 5′-CACATCCTCATCCTCGTCATTC -3′ (reverse) |
| Human ABCG1 | 5′-ATTCAGGG ACCTTTCCTATTCGG-3′ (forward) |
|  | 5′-CTCACCACTATTGAACTTCCCG-3′ (reverse) |
| Mouse ABCG1 | 5′-CTTTCCTACTCTGTACCCGAGG-3′ (forward) |
|  | 5′-CGGGGCATTCCATTGATAAGG-3′ (reverse) |
| Human GAPDH | 5′-TGTGGGCATC AATGGATTTGG-3′ (forward) |
|  | 5′-ACACCATGTATTCCGG GTCAAT-3′ (reverse) |
| Mouse GAPDH | 5′-GGTGTGAACCATGAGAAGTATGA-3′ (forward) |
|  | 5′-GAGTCCTTCCACGATACCAAAG-3′ (reverse) |
